# Efficacy of Sulforaphane in Neurodegenerative Diseases

**DOI:** 10.3390/ijms21228637

**Published:** 2020-11-16

**Authors:** Giovanni Schepici, Placido Bramanti, Emanuela Mazzon

**Affiliations:** IRCCS Centro Neurolesi “Bonino-Pulejo”, Via Provinciale Palermo, Contrada Casazza, 98124 Messina, Italy; giovanni.schepici@irccsme.it (G.S.); placido.bramanti@irccsme.it (P.B.)

**Keywords:** sulforaphane, neuroprotective effects, Alzheimer’s disease, Parkinson’s disease, multiple sclerosis

## Abstract

Sulforaphane (SFN) is a phytocompound belonging to the isothiocyanate family. Although it was also found in seeds and mature plants, SFN is mainly present in sprouts of many cruciferous vegetables, including cabbage, broccoli, cauliflower, and Brussels sprouts. SFN is produced by the conversion of glucoraphanin through the enzyme myrosinase, which leads to the formation of this isothiocyanate. SFN is especially characterized by antioxidant, anti-inflammatory, and anti-apoptotic properties, and for this reason, it aroused the interest of researchers. The aim of this review is to summarize the experimental studies present on Pubmed that report the efficacy of SFN in the treatment of neurodegenerative disease, including Alzheimer’s disease (AD), Parkinson’s disease (PD), and multiple sclerosis (MS). Therefore, thanks to its beneficial effects, SFN could be useful as a supplement to counteracting neurodegenerative diseases.

## 1. Introduction

Sulforaphane (SFN) known as [1-isothiocyanato-4-(methylsulfinyl)butane], is an aliphatic isothiocyanate whose precursor, the glucoraphanin, is mainly found in cruciferous vegetables such as broccoli, cauliflower, cabbage, and Brussels sprouts [1]. SFN is produced by the degradation of glucosinolates, compounds that are biologically inactive, characterized by a basic structure consisting of β-D-thioglucose, an oxime sulfonate, and a variable side chain constituted of amino acids including methionine, tryptophan, phenylalanine, or other amino acids [2]. Moreover, the side chain structure can contain aliphatic, aromatic, or heterocyclic groups that determine the chemical, physical, and biological properties of the isothiocyanates [3]. The biosynthesis of SFN occurs through a hydrolysis reaction that involves the myrosinase enzyme, present in plants, which together with the inactive form of epithiospecifier (ESP) protein leads to the formation of SFN. Conversely, the active form of ESP together with myrosinase leads to the synthesis of SFN nitrile [4,5].

Thanks to its molecular structure and lipophilic nature, SFN shows a high bioavailability. Indeed, it was demonstrated that SFN in rats reaches the plasma peak in 4 h, with an average half-life of about 2.2 h [6,7]. Instead, in mammals, SFN is rapidly metabolized through a conjugation reaction catalyzed by glutathione (GSH) S-transferase that involves GSH and leads to the formation of a concentration gradient through continuous absorption of SFN, which balances its export [4,8]. Moreover, the SFN concentration gradient appears to involve P-glycoprotein and Multidrug Resistance Associated Protein 1 important in membrane transport [9].

**Figure 1:**
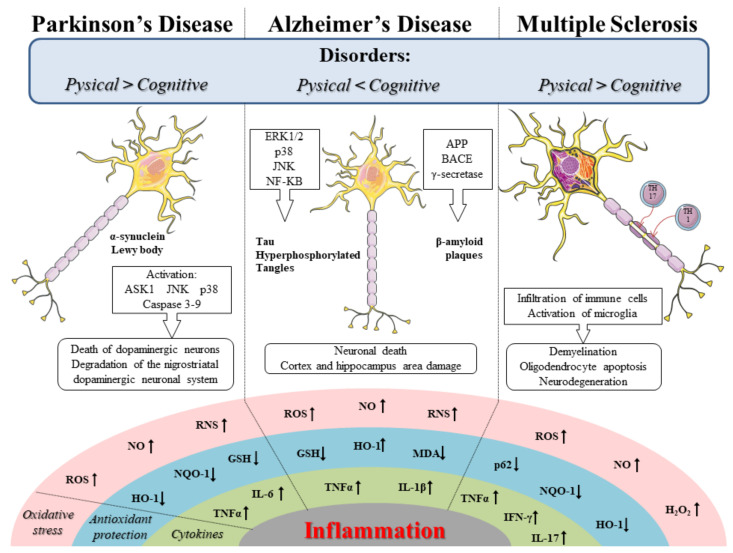
Schematic representation of the oxidative stress and inflammation pathways in Parkinson’s disease (PD), Alzheimer’s disease (AD), and multiple sclerosis (MS). The figure was made taking the images from Servier Medical Art (available at http://smart.servier.com/), licensed under a Creative Commons Attribution 3.0 Unported License (https://creativecommons.org/licenses/by/3.0/). ROS, Reactive oxygen species; NO, Nitric oxide; RNS, Reactive nitrogen species; HO-1, Heme oxygenase 1; NQO-1, Nicotinamide adenine dinucleotide phosphate quinone oxidoreductase 1; GSH, Glutathione; MDA malondialdehyde; p62; H_2_O_2,_ Hydrogen peroxide; TNF-α, Tumor necrosis factor-α; IL-6, Interleukin-6; IL-1β, Interleukin-1β; IFN-γ, Interferon- γ; ERK, Extracellular signal-related kinase; p38; JNK, c-Jun N-terminal kinase; NF-κB, Nuclear factor kappa-B; ASK1, Apoptosis signal-regulating kinase 1; APP, Amyloid precursor protein; Aβ, Beta-amyloid; BACE, β-secretase.

SFN is a phytocompound with antioxidant, anti-inflammatory, and antiapoptotic effects. Since oxidative stress, inflammation, and mitochondrial dysfunction are involved in neurodegenerative diseases, the interest of the scientific community for it has grown. Therefore, based on its properties, SFN was investigated in main neurodegenerative diseases including Alzheimer’s disease (AD), Parkinson’s disease (PD), and multiple sclerosis (MS). In Figure 1, we schematize the oxidative stress and inflammation pathways involved in the PD, AD, and MS.

The aim of our work is to demonstrate the efficacy of SFN in neurodegenerative diseases including AD, PD, and MS. In order to write this review, we performed a PubMed search, using the keywords: “sulforaphane” and “Alzheimer’s disease” (35 articles), “sulforaphane” and “Parkinson’s disease” (33 articles), and “sulforaphane” and “Multiple sclerosis” (13 articles). We considered the articles published between 2005 and 2020 that reported the effects of SFN in the management of neurodegenerative diseases.

## 2. SFN and Its Molecular Target

SFN is a sulfur-rich compound, with molecular target nuclear factor erythroid 2 related factor 2 (Nrf2). Moreover, several pathological events occurring the neurodegenerative diseases such as neuroinflammation, oxidative stress, mitochondrial dysfunction, excitotoxicity, and neuronal damage, can involve Nrf2 [8]. Indeed, Nrf2 expression can be regulated by SFN, that acting through cytoprotective enzymes with detoxifying and antioxidant action, such as nicotinamide adenine dinucleotide phosphate quinone oxidoreductase 1 (NQO-1), heme oxygenase 1 (HO-1), GSH S-transferase, and thioredoxin reductase, thus counteract the oxidative stress [10]. Nrf2 is a basic transcription factor leucine zippered (bZIP) with a cap and collar structure, encoded in humans by *NFE2L2* gene. In addition, Nrf2 represents the main regulator of redox balance and cellular detoxification responses, it also stimulates the cellular defense mechanism [11]. Nrf2 signaling pathway involves the antioxidant response elements (ARE) and Kelch-like ECH associated protein 1 (Keap1). In physiological conditions, Keap1 sequestrates Nrf2 in the cytoplasm, which acts as a sensor redox thanks to the cysteine residues, to facilitate the ubiquitination and degradation of Nrf2 through the ubiquitin-proteasome system (UPS) [12,13]. Subsequently, Nrf2 is translocated into the nucleus where the oxidation occurs of the cysteine residues of Keap1 and the subsequent its stabilization [14,15,16]. In the nucleus, Nrf2 by interacting with musculoaponeurotic fibrosarcoma oncogene homologue (Maf), activates ARE, which plays a key role in the transcriptional regulation of cytoprotective genes, thus exerting a neuroprotective action [17]. Instead, in stress conditions such as oxidative stress and production of reactive oxygen species (ROS) or reactive nitrogen species (RNS), the Nrf2 ubiquitination and degradation are blocked [13]. Therefore, SFN through Keap1/Nrf2/ARE pathway and in particular by enhancing the Nrf2 activation, can contribute to counteract the oxidative stress through the upregulation of molecules such as GSH peroxidase 1, NQO-1, HO-1, and gamma-glutamylcysteine synthetase, which controls the GSH synthesis [18,19].

The neuroprotective role of SFN is correlated with the Nrf2 pathway; in fact, it was shown that in mice the Nrf2 knockout (KO), or the combined treatment of SFN with gamma-glutamylcysteine synthetase inhibitors, abolished the neuroprotective effects of SFN, thus confirming that SFN targets Nrf2 [20,21,22]. In addition, SFN can also exert anti-inflammatory effects, reducing the neuronal damage mediated by microglial activation and regulating the levels of inflammatory mediators, such as tumor necrosis factor-α (TNF-α), interleukin (IL) 6, IL-1β, inducible nitric oxide synthetase (iNOS), and cyclooxygenase-2 (COX-2) [23,24]. In the same way, SFN can lead to a decrease of mitogen-activated protein kinases (MAPK) including p38, extracellular signal-regulated kinase (ERK) 1/2, and c-Jun N-terminal kinase (JNK), as well as others inflammatory mediators such as Receptor Interacting Serine/Threonine Kinase 3, mixed lineage kinase domain-like, and nuclear factor kappa-B (NF-κB), to lead to the reduction of necrosis and neuronal apoptosis [25]. In the same way, SNF can also reduce the cleavage of caspase-1 and caspase-3, involved respectively in inflammation and apoptosis, as well as increase the release of anti-inflammatory cytokines such as IL-4 and IL-10, to favor the reduction of neuroinflammation and cell death [26].

Moreover, SFN through the formation of oxidative stress can promote autophagy in neurons, probably according to a mechanism that involves the Nrf2 pathway, as shown in Nrf2-KO mice, that instead expressed a lower number of genes related to autophagy [27]. Although, a further study excluded the involvement of Nrf2 in autophagy [28]. Indeed, it was shown that SFN through a mechanism that involves ERK induces the increase in the levels of light chain 3-II (LC3-II), a protein involved in the assembly to autophagosomal membranes [28,29].

SFN can also exert a neuroprotective action on mitochondrial function; since neurons are metabolically active to meet energy requirements, they need healthy mitochondria [30]. Therefore, SFN through Nrf2 pathway can promote the activation of genes that favor the mitochondrial biogenesis, thus preserving the mitochondrial complex I, II, and IV, to lead to the production of adenosine triphosphate (ATP), otherwise reduced by neuronal damage [31].

Furthermore, SFN can also promote neurogenesis, increasing the levels of brain-derived neurotrophic factor (BDNF) and upregulating the expression of key proteins in the Wingless type (WNT) signaling pathway, including β-catenin and cyclin D1, involved in proliferation and differentiation neuronal [32].

## 3. Effects of Sulforaphane in Experimental Studies of Alzheimer’s Disease

AD is a chronic neurodegenerative disease characterized by progressive cognitive deficits [33]. The disease shows extracellular aggregates of beta-amyloid (Aβ) peptides leading to the formation of plaques and intracellular accumulation of hyperphosphorylated tau protein, which in turn leads to the formation of neurofibrillary tangles [34,35]. In particular, the peptides Aβ_1–40_ and Aβ_1–42_ generated by the proteolytic cleavage of the amyloid precursor protein (APP) are responsible of pathological events of the disease such as oxidative damage, inflammation, and increase of intracellular calcium [36]. Indeed, it was also shown that increase of oxidative stress and reduced folding of endoplasmic reticulum proteins, as well as autophagy, accelerated the Aβ aggregates and tau protein levels [37]. Therefore, new strategies capable to block the neurodegeneration and counteract the disease to support those already known such as SFN are needed [38].

### 3.1. Effects of Sulforaphane in In Vivo AD Models

The effects of SFN were shown by Hou et al. in PS1V97L transgenic mice administered via intraperitoneal with SFN (5 mg/kg) for four months and compared to the control mice. It was demonstrated that SFN preserved the animals from cognitive deficits evaluated through behavioral tests. Moreover, it was shown that SFN inhibited Aβ aggregation, tau hyperphosphorylation, as well as oxidative stress, evaluated through GSH and malondialdehyde (MDA) levels and also neuroinflammation assessed by TNF-α and IL-1β levels. To confirm the results obtained in vivo, the authors performed the study also in the primary cortical cells of rats. In addition, it was observed that SFN (0.1 μM) improved the viability as well as preserved the dendritic length of the cortical neurons, which was reduced following Aβ oligomers incubation at different concentrations of 5 µM, 10 µM, and 20 µM. Therefore, the results of the study demonstrate that SFN can be useful to counteract the Aβ aggregation in AD [39]. In addition, the authors demonstrated the effects of SFN in Wang et al. in Sprague-Dawley male rats treated with intracerebroventricular injections of Aβ (10 µL). Indeed, it was shown that the treatment via intraperitoneal with SFN (5 mg/kg) for seven days improved depression behavior and spatial learning. SFN likely preserved the depressive behavior, through the serotonergic system, by modulating both the activity of the enzyme tryptophan hydroxylase, involved in serotonin metabolism and serotonin transporter. In the same way, it was shown that in rat brains, SFN reduced the neuroinflammation and oxidative stress, respectively measured through the reduction of levels of MDA, TNF-α, and IL-1β, as well as by the increase of GSH [40]. Zhang et al. also demonstrated the effects of SFN (25 mg/kg) administered orally, in C57BL/6 mice with Alzheimer-like lesions induced by the combined treatment of D-galactose (200 mg/kg), via subcutaneous and aluminum chloride dissolved in water (0.4 g/100 mL). The authors showed that SFN treatment improved cognitive and locomotor deficits evaluated by Morris water maze and open field test. Moreover, it was reported that SFN protected against the formation of Aβ plaques in the cortex and hippocampus, reduced by the increase of oxidative stress [41]. Zhang et al. in another study showed the effects of SFN in AD-like mice. To induce the model eight-week-old Kunming mice were treated with aluminum (20 mg/kg) via intragastric and D-galactose (120 mg/kg) injected subcutaneously. The combined administration of aluminum and galactose produced neurotoxicity, mimicking the aging of mice, leading to learning and memory deficits. In addition, it was found that in neurons, aluminum can induce the APP overexpression and consequently increase Aβ production. Moreover, it was demonstrated that the daily SFN (25 mg/kg) treatment via gavage for 90 days improved the cognitive deficits as well as decreased the loss of cholinergic neurons, evaluated through the reduction of aluminum levels in the hippocampus and medial septum. However, the spectrophotometric analysis did not show significant differences either between the level of acetylcholine and acetylcholinesterase activity in the cortex of experimental groups treated with SFN and not treated compared to the control group. Consequently, the reasons for these results remain unclear and therefore are needed further studies [42].

In addition, Kim et al. reported the effects of SFN in AD mice. To induce the model, ICR mice were administered in acute with a single intracerebroventricular injection of Aβ (5 µL). It was shown that the treatment intraperitoneal with SFN (30 mg/kg) for six days improved the cognitive and memory deficits. However, SFN did not prevent the formation of aggregated Aβ, and treatment with this isothiocyanate reduced the oxidative stress, likely through a mechanism that involved the proteasomal activation. Therefore, SFN can be a candidate for counteract the oxidative stress in AD [43]. The oxidative stress is a mechanism that involves the Nrf2/ARE pathway. Indeed, a reduction of Nrf2 expression was observed in AD [44]. In addition, Nrf2 lack leads to increase of oxidative stress, as well as an increase in autophagic dysfunction and tau phosphorylation [45,46]. Conversely, Nrf2 upregulation favors the neuroprotection against the oxidative stress [44,47]. Nrf2 plays the role of transcriptional activator by binding to the ARE site, modulating the expression of target genes. Indeed, Nrf2/ARE regulates the expression of β-secretase 1 (*BACE-1*) involved in Aβ aggregation and consequently in AD [48]. Moreover, a recent study demonstrated that SFN, according to a dose-dependent mechanism, can inhibit BACE-1 and consequently Aβ aggregation in a more powerful way compared to quercetin and resveratrol used like controls [49].

Lee et al. have also demonstrated the effects of SFN (10 or 50 mg kg^−1^) administered via oral gavage, in transgenic 3 × Tg-AD mice. The SFN treatment improved learning and memory deficits. Moreover, the subsequent immunohistochemical analysis showed that SFN significantly reduced the Aβ levels in the cortex, as well as Aβ and tau levels in the hippocampus. Likewise, it was also reported that SFN increased the levels of co-chaperone of heat shock protein (HSP), C-terminus of HSP 70-interacting protein (CHIP), a protein highly expressed in the brain and involved in ubiquitination and degradation of Aβ, tau, and BACE-1. In order to confirm these results, the experiment was also conducted in CHIP-deficient neurons derived from 3 × Tg-AD. Indeed, the treatment with SFN increased the level of CHIP reducing also the Aβ and tau levels, thus avoiding their aggregation and confirming the results obtained in vivo. Therefore, SFN modulating CHIP could be useful to counteract the cellular stress and protein misfolding that characterize the neurodegenerative diseases such as AD [50].

The antioxidant and anti-inflammatory effects of SFN were also reported in the oxidative stress associated with type 2 diabetes mellitus, which could contribute to cognitive deficits and AD. Indeed, it was also shown that Aβ plaques and neurofibrillary tangles were found in the postmortem brain of diabetic patients [51]. Moreover, Aβ aggregates were found in the pancreatic cells of diabetic patients, thus favoring the loss of beta cells and the progression of diabetes. Consequently, AD and diabetes could show common pathological signs [52,53]. In the latter, Pu et al. demonstrated in type 2 diabetes mellitus transgenic mice that SFN (1 mg/kg) administered via intraperitoneal for 28 days improved the cognitive deficits compared to the control mice. In addition, the immunohistochemical and Western blotting analysis reported both a reduction of Aβ plaques and phosphorylated tau levels in the hippocampus. SFN can probably exert its beneficial effects in the brain through the Nrf2 overexpression, enhancing the antioxidant action of HO-1 and NQO-1, thus leading to the reduction of oxidative stress [54] (see Table 1).

### 3.2. Effects of Sulforaphane in In Vitro AD Models

In order to investigate the correlation between Nrf2 and AD, Bahn et al. showed the effects of SFN in vitro and in vivo. The authors demonstrated that SFN (1 μM), in SH-SY5Y cells, led to the overexpression of Nrf2 and reduced the transcription level of *BACE1* and *BACE1-AS*, involved in amyloidogenesis processes. In addition, SFN treatment led to modulation of HO-1 levels involved in the oxidative stress, and also the reduction of Aβ aggregation. On the contrary, Nrf2 inhibition led to an overexpression of Keap1, increasing the transcription level of *BACE1* and *BACE1-AS* and consequently of Aβ formation. In the same way, the study was conducted in vivo using 5xFAD mice, a model that reproduces severe AD, and in 3 × Tg-AD mice that instead show Aβ aggregates and tau phosphorylated. Indeed, both in 5xFAD mice and in 3 × Tg-AD mice, it was demonstrated that SFN (5 or 10 mg/kg) administered via intraperitoneal, probably through Nrf2, improved the cognitive deficits, reduced BACE-1 expression, and Aβ aggregation. Additionally, immunohistochemical analysis has shown that in 3 × Tg-AD mice, SFN reduced the phosphorylated tau levels, according to a dose-dependent mechanism. Therefore, the study confirmed that one possible strategy to counteract the AD could be represented by the mechanism that involves Nrf2 activators such as SFN [48].

The antioxidant effects of SFN, as well as its interaction with Nrf2, were shown by Masci et al. in an in vitro AD model. The authors investigated the neuroprotective effects of two crude juices of broccoli sprouts (10 μM), grown in the presence of sucrose and exposed to dark or white light, to distinguish them for the content of phenols and anthocyanin. In order to induce the model, human neuroblastoma SH-SY5Y cells were treated with Aβ_25–35_ (25 μM) and juices of broccoli sprouts. The results of the study demonstrated that treatment with both juices exerted a protective action against the cytotoxicity and cell death Aβ induced, as shown by the overexpression of HSP70, a molecular chaperone known to protect the cell from oxidative stress. Moreover, the authors were also demonstrated a reduction of oxidative stress evaluated by intracellular increase of GSH, as well as HO-1 and NQO-1 activity likely induced through the Nrf2 signaling pathway. It is noteworthy that although both juices showed similar effects, the juice with a higher polyphenol composition was more powerful in the Nrf2 activation [55]. In addition, Lee et al. demonstrated the effects of SFN in SH-SY5Y cells induced with Aβ_25–35_ (15 μM). It has been demonstrated that the SFN (1 μM, 2 μM, and 5 μM) pretreatment protected the cells from the cytotoxicity and apoptosis, according to a dose-dependent mechanism. Indeed, SFN reduced apoptosis by upregulating B cell lymphoma-2 (BCL-2), as well as reducing the activation of JNK and proapoptotic protein BCL2-Associated X (BAX). Moreover, it was observed that anti-apoptotic activity of SFN appeared to be mediated by oxidative stress, as demonstrated by increased the antioxidant enzymes such as NQO-1, HO-1, and gamma-glutamylcysteine synthetase. In the same way, it was also demonstrated that SFN exerts its antiapoptiotic effect through Nrf2. Indeed, the Nrf2 block abolished the beneficial effects of SFN. Therefore, the results obtained reported that activation of the Nrf2 pathway could be an accessible way to counteract the disease [56].

Microglial cells known for the neuroinflammatory functions were studied in AD to eliminate harmful Aβ aggregates through phagocytosis and also to the release of pro-inflammatory cytokines. Therefore, a useful strategy to control the Aβ aggregates can be to exploit the phagocytic activity of microglial cells without triggering a pro-inflammatory response [57]. Chilakala et al. showed, in an in vitro study, the effects of SFN (5 µM), in microglial cells pretreated with Aβ oligomers (100 ng/mL, 500 ng/mL, and 1000 ng/mL). The study reported that microglial cells show poor phagocytic activity at low Aβ oligomers concentrations. Furthermore, it was shown that in microglial cells with high Aβ oligomers concentrations cause toxicity, leading to the modulation of genes that regulate phagocytosis. Consequently, both at low and high concentrations of Aβ oligomers, the treatment with SFN (5 µM) induced an increase of phagocytic activity. In addition, it was also shown that at low-dose Aβ oligomers, SFN did not promote the release of pro-inflammatory microglia mediators. Therefore, the study demonstrated that SFN can improve the microglial phagocytosis, reduced due to Aβ aggregates [58]. Since the microglial activation plays a key role in neuroinflammation, anti-inflammatory strategies are needed that are capable of counteracting the diseases that involve the central nervous system (CNS) such as AD [59]. Subedi et al. showed, both in vitro and in vivo, the effects of SFN-enriched broccoli sprouts, obtained through pulsed electric field exposure to increases the glucosinolate production and myrosinase activity, as well as increased the SFN level. The SFN level was obtained by high-performance liquid chromatography and the injection volume was 10 μL. In order to show the effects of SFN, murine microglia cells and neuroblastoma cells were used. Indeed, it was demonstrated that SFN treatment reduced inflammation and improved cell viability through a mechanism that down-regulated the MAPK signaling, involved in cellular responses. Moreover, it was also shown that SFN inhibited the inflammation, following the microglial activation induced by lipopolysaccharide (100 ng/mL). Likewise, it was reported that SFN upregulated Nrf2 and HO-1, reducing oxidative stress and apoptosis. In addition, it was demonstrated that in ICR mice, the administration via oral of broccoli sprout (200 mg/kg) improved the memory deficits induced by intraperitoneal scopolamine treatment (1.2 mg/kg), likely through the activation of Nrf2 mediated by SFN; in particular, through the mechanism that involved the inhibition of caspase-3, and thus exerting a neuroprotective action [60]. Moreover, Zhao et al. showed in a cellular model of AD how SFN through Nrf2 can exert its effects. Neuroblastoma N2a cells harboring human mutant amyloid precursor protein were treated with SFN (1.25 and 2.5 μM), for 48 h and compared to the control group. The treatment with SFN reduced the intracellular levels of Aβ_1–40_ and Aβ_1–42_ in proportion to the increase of SFN concentration. Moreover, SFN led to a reduction of oxidative stress evaluated through the decrease of MDA and by the increase of superoxide dismutase (SOD). Moreover, it was also demonstrated that SFN led to a reduction of the inflammation evaluated through the decrease of proinflammatory cytokines. Therefore, the study confirmed that SFN performs its anti-inflammatory effect through Nrf2 upregulation [61]. The anti-inflammatory effect of SFN on Aβ monomers, as well as the mechanism underlying this effect, was investigated by An et al. in human THP-1 macrophages induced with Aβ_1–42_ (10 or 20 μM). The treatment with SFN (5 μM) inhibited the activation of the inflammasome Nod-Like Receptor Protein 3 dependent on both caspase-1 and cathepsin B. Moreover, SFN has also reduced the secretion of IL-1β, through a mechanism that involved the dephosphorylation of signal transducer and activator of transcription 1 and consequently also activated the Nrf2/HO-1 pathway. Similarly, the mechanism underlying the anti-inflammatory effect of SFN was confirmed by the herbimycin A (1 μM) pretreatment, that like SFN reduced the activation of signal transducer and activator of transcription 1 induced by Aβ_1–42_. Therefore, SFN could be useful to reduce the neuroinflammation in AD [62]. The chronic inflammatory process that occurs in AD could also lead to the dysregulation of Mer tyrosine kinase, regulated by Aβ and involved in phagocytosis and cytokine production [63]. Although the mechanism is not fully understood, Jhang et al. showed how SFN modulated Mer tyrosine kinase expression in human THP-1 macrophages. The authors reported that induction of Aβ_1–42_ (5, 10 and 20 μM) could downregulate the level of Mer tyrosine kinase, favoring the intracellular increase of calcium and NF-κB activation, with a consequent excessive production of IL-1β and TNF-α, which in turn could act as negative regulators of Mer tyrosine kinase expression. Conversely, the pretreatment with SFN (5 μM) has significantly reversed the effects of Aβ_1–42_. Moreover, it was shown that Mer tyrosine kinase block, reduced the anti-inflammatory effect of SFN. Therefore, SFN, acting through Mer tyrosine kinase could exert an anti-inflammatory effect against the neuroinflammation induced by Aβ_1–42_, thus proving to be a useful strategy for counteract the disease [64].

SFN can also exert protective effects through BDNF, involved in cell survival and synaptic plasticity. Kim et al. demonstrated both in vitro and in vivo the effects of SFN. In vitro, it was shown in cortical neurons of ICR mice, that SFN (10 or 20 μM), increased the BDNF expression. Moreover, it was also demonstrated that SFN, increased the levels of postsynaptic density protein 95, microtubule-associated protein 2, and synaptophysin, as well as activated the components of tropomyosin-related kinases B signaling. In the same way, the results obtained in vitro, were confirmed in vivo; in fact, it was shown that in 3 × Tg-AD mice the SFN (10 or 50 mg/kg/day) treatment via gavage for eight weeks determined an increase of BDNF levels in the cortex and hippocampus. Moreover, SFN also increased the levels of presynaptic proteins such as synaptophysin, microtubule-associated protein 2, and postsynaptic density protein 95. Noteworthily, as in the cortex, SFN treatment also activated the components of tropomyosin-related kinases B signaling, thus promoting the neuronal survival. Therefore, it was confirmed that SFN can play its action through BDNF, as shown by the reduction of histone acetylation of the BDNF promoter regions, as well as by the increased of its expression levels [65].

In addition, in brains of AD patients, a reduction in the expression levels of the p75 neurotrophin receptor (p75^NTR^) was also demonstrated [66,67,68]. SFN can also modulate the expression of p75^NTR^, which plays a key role in the pathogenesis of AD [69]. Indeed, it was observed that the deletion of the *p75^NTR^* gene favored the increase of Aβ aggregates [70]. Therefore, the upregulation of *p75^NTR^* could be a useful therapeutic strategy to reduce the formation of Aβ aggregates in the brain. Zhang et al. investigated, both in vitro and in vivo, if the neuroprotective effects of SFN were mediated by the upregulation of p75^NTR^. Human SH-SY5Y neuroblastoma cells were pretreated with SFN (2 μM) and subsequently exposed to different Aβ concentrations (10–80 μM). The treatment with SFN prevented the reduction of cell viability induced by Aβ (40 and 80 μM). In addition, in SH-SY5Y cells, the SFN treatment preserved both the reduction of mRNA and expression level of p75^NTR^ protein. Therefore, it was demonstrated that SFN can exert its effect also through the upregulation of *p75^NTR^*, by the silencing of histone deacetylases 1 and 3. To confirm the results obtained in vitro, the study was performed in vivo. Indeed, it was demonstrated that the administration of SFN (25 mg/kg), via gavage in APP/presenilin1 double transgenic mouse, improved the cognitive deficits and preserved the Aβ plaques increase in the cortex of the animals. Moreover, the Western Blot and polymerase chain reaction analysis showed that in the cortex, SFN preserved by the reduction of mRNA and p75^NTR^ protein expression. Therefore, the study demonstrated that SFN by upregulating p75^NTR^ and regulating histone acetylation can avoid Aβ aggregation and thus exert a beneficial effect in AD [71] (see Table 2).

## 4. Effects of Sulforaphane in Experimental Studies of Parkinson’s Disease

PD is a neurodegenerative disease characterized by cognitive and motor deficits such as bradykinesia, muscle stiffness, and tremor. The disease involves the progressive loss of dopaminergic neurons in the substantia nigra compacta and also α-synuclein inclusions known as Lewy bodies [33]. Several harmful events such as oxidative stress and a loss of GSH from the substantia nigra were identified in the postmortem brain of PD patients [72,73]. In fact, the oxidative stress is a pathological alteration that increases the vulnerability of dopaminergic neurons through stimuli such as aging and exposure to neurotoxic compounds that promote apoptosis and death of dopaminergic neurons [74]. Therefore, both oxidative stress and a loss of GSH are events known to induce the Nrf2 activity, as shown by the increase of its nuclear level in neurons contained in substantia nigra of the postmortem brain of AD patients, as well as by the presence aberrant of Keap1 in Lewy bodies [75,76]. In order to confirm the induction of Nrf2 activity in PD, it was also demonstrated that the expression of some proteins regulated by Nrf2 was dysregulated. Indeed, in the brain of PD patients, high levels of NQO-1 and HO-1 were found [77]. Since the endogenous expression of Nrf2 is not able to prevent cell death, a strategy based on Nrf2 activators compounds like SFN could be useful to prevent the neurodegeneration and counteract the disease [78]. Several experimental studies both in vitro and in vivo demonstrated the efficacy of SFN in PD.

### 4.1. Effects of Sulforaphane in In Vitro PD Models

Niso-Santano et al. investigated in vitro the role of Nrf2 in the PD. In order to induce the PD model, SH-SY5Y human neuroblastoma cells were treated with paraquat in a concentration range (0–100 µM) for 24 h. The induction of the model led to an increase of ROS and cell death, related to the activation of apoptosis signal-regulating kinase 1 (ASK1 also known as MAP3K5), as well as in the activation of JNK and p38. By contrast, it was demonstrated that SFN (5 µM) pretreatment or vitamin E before to induce the model blocked the ASK1 signaling pathway and prevented both oxidative stress and cell death. Since in PD the treatment with vitamin E showed conflicting results, the authors evaluated, through the modulation of thioredoxin levels, the antioxidant capacity in mouse embryonic fibroblasts and SH-SY5Y cells. It is noteworthy that Nrf2 modulated the gene expression of thioredoxin; indeed, undetectable thioredoxin levels in fibroblasts Nrf2-deficient were shown. On the contrary, it was also demonstrated in SH-SY5Y cells pretreated with SFN (5 µM) and fibroblasts Keap1-deficient both high levels of thioredoxin and a reduction of cell death. Therefore, the study identified the Nrf2/thioredoxin axis as a useful therapeutic target for counteracting the oxidative stress and neurodegeneration in PD [79]. Bao et al. showed the effect of SFN in PC12 undifferentiated cells, induced with 1-methyl-4-phenyl pyridine (MPP^+^), which represents the toxic oxidized species of 1-methyl-4-phenyl-1,2,3,6-tetrahydropyridine (MPTP). In order to induce the model, PC12 cells were incubated with MPP^+^ (100, 300, 500, and 700 µmol/L) for different time periods (12, 24, and 48 h), as well as evaluated the cell viability and apoptosis. The pretreatment with SFN (0.5, 1.0, 2.5, 5.0, and 10 µmol/L) for one hour, before of the exposure for 24 with MPP^+^ (500 µmol/L) has significantly attenuated the cell damage induced by oxidative stress. Indeed, SFN exerted this protective effect, probably through the upregulation of Nrf2-ARE pathway, as shown by the reversion in the reduction of HO-1 and NQO-1 expression, which was reduced following at MPP^+^. Consequently, SFN has also reduced the oxidative stress and prevented the cell damage [80]. The neuroprotective effects of SFN were also reported by Deng et al. in PC12 cells induced with 6-hydroxydopamine (6-OHDA) (80 µM) for 24 h. SFN (0.1, 1, and 5 µM) pretreatment for 6 h led to an increase in cell viability and inhibited cell death 6-OHDA-induced in a dose-dependent manner. The authors also showed the cytoprotective effects of SFN by evaluating the expression of molecules involved with endoplasmic reticulum stress. Since the endoplasmic reticulum is important in the processing and folding of proteins, as well as in the calcium signaling, it was demonstrated that in PD, the oxidative stress can lead to the alteration of the endoplasmic reticulum function and also in the signaling pathways their associated. Indeed, under stress conditions, it was demonstrated that in the endoplasmic reticulum, both alterations occur in calcium homeostasis and the accumulation of misfolded proteins, with consequent activation of unfolded protein response pathway. In addition, the phosphorylation of the eukaryotic initiation factor 2α was found to involve the reduction of protein synthesis. Likewise, the activation of molecular chaperones such as Binding Immunoglobulin Protein was shown, involved in the correct protein folding. However, whether the function of the endoplasmic reticulum is severely impaired, the apoptotic pathway is activated, as shown by the increase in C/EBP homologous protein. Instead, the treatment with SFN (5 µM) has shown a cytoprotective role by reducing the stress in the endoplasmic reticulum, as reported by the expression levels of Binding Immunoglobulin Protein and C/EBP homologous protein, both altered after 6-OHDA induction. In the same way, it was shown that SFN can act in the stress applied to the endoplasmic reticulum via Nrf2. Indeed, it was demonstrated that the Nrf2 block prevented the cytoprotective effects of SFN in PC12 cells 6-OHDA-induced [81]. In addition, the same research group showed the effects of SFN in PC12 cells induced with 6-OHDA (40 or 80 µM) for 24 h. SFN (1 and 5 µM) pretreatment for 24 h prevented the cell damage, evaluated through cell viability and also by decreasing of caspase-3 activity. Moreover, it was demonstrated that SFN exerts its cytoprotective effect through the activation and subsequent translocation of Nrf2 into the nucleus with a consequent increase of HO-1 expression level, mediated by the activation of phosphatidylinositol-3-kinase (PI3K)/AKT pathway. Confirmation of PI3K/Akt activation by SFN was demonstrated in PC12 cell pretreated with LY294002, an inhibitor of the PI3K/Akt pathway, which blocked the beneficial effects of SFN, as well as Nrf2 translocation into the nucleus and also the increased of HO-1 expression levels. Therefore, these results suggest that SFN, through mechanisms that involve Nrf2 activation, can play a protective effect for counteracting the neurodegeneration that occurs in the PD [82]. Oxidative stress and inflammation are key factors in the progression of PD; consequently, strategies to increase Nrf2 activity can be useful to counteracting the oxidative stress and neurodegeneration [83]. Siebert et al. demonstrated the effects of Nrf2 activators, SFN, and tert-butylhydroquinone in the dopaminergic neurons of organotypic rat nigrostriatal cultures, induced with 6-OHDA (100 nM). Organotypic Cultures were treated with SFN (5 μM) for 48 h before the combined treatment with SFN (5 μM) and 6-OHDA (100 nM) for 16 h. In the same way, the effects of tert-butylhydroquinone (50 μM) were tested for 8 h prior to the combined treatment of tert-butylhydroquinone (50 μM) and 6-OHDA (100 nM) for a further 16 h. The immunohistochemical analysis showed that both SFN and tert-butylhydroquinone reduced the nigrostriatal neurodegeneration induced by 6-OHDA. Moreover, it was shown that the combined treatment of SFN and tert-butylhydroquinone has almost completely preserved the nigrostriatal neurons by neurodegeneration, probably through a mechanism mediated by the increase in the activity of antioxidant enzymes like NQO-1. Therefore, Nrf2 activators such as SFN may be useful to counteracting the degeneration of dopaminergic neurons in PD [84]. Izumi et al. showed, in PC12 cells induced with 6-OHDA (250 µM) for 24 h and also in primary mesencephalic cultures, the protective effects of TPNA10168, an a Nrf2 activator. The treatment with TPNA10168 (0.1–30 µM) in PC12 cells demonstrated a neuroprotective effect through NQO-1 upregulation. Similarly, it was also shown that in primary mesencephalic cultures, TPNA10168 upregulated HO-1 in astrocytes, thereby protecting dopaminergic neurons from neurodegeneration. Moreover, TPNA10168 demonstrated greater efficacy in activating Nrf2 and also thus a better antioxidant action, as well as lower cytotoxicity compared to SFN (0.1–10 µM) [85]. Vauzour et al. showed the neuroprotective effects of SFN in primary cortical neurons of mouse, induced with 5-S-Cysteinyl-dopamine (100 μM), a dopamine oxidation metabolite also found in PD patients. SFN (0.01–1 μM) pretreatment for 24 h protected neurons by cell death 5-S-Cysteinyl-dopamine-induced. In addition, it was shown that SFN apart from inducing the translocation of Nrf2 and the consequent increase in activity of antioxidant enzymes such as NQO-1 and GSH-S-transferase, it also seems to implicate activation of the ERK 1/2 and protein kinase B pathways, involved in survival and growth cell [86]. The activation of antioxidant enzymes such as NQO-1 is crucial to counteracting oxidative stress, as well as the consequent loss of dopaminergic neurons. Therefore, potent NQO-1 activators such as SFN are useful for avoiding the dopamine oxidation to dopamine quinone and subsequent neuronal vulnerability [87,88]. Han et al. showed the effects of SFN (0.5, 1, 2.5, and 5 μM) in dopaminergic cell lines CATH.a and SK-N-BE (2) C and in mesencephalic dopaminergic neurons 6-OHDA induced with (50 μM) or tetrahydrobiopterin (200 μM). The pretreatment with SFN preserved dopaminergic neurons and mesencephalic neurons from the neurodegeneration induced by 6-OHDA and tetrahydrobiopterin, thus avoiding the production of dopamine quinone, oxidative stress, and neuronal damage. Moreover, it was also shown that SFN treatment has avoided both ROS production and membrane damage. Consequently, SFN favored the increase of NQO-1 activity, leading to the intracellular removal of dopamine quinone and thus avoiding the neurodegeneration [89]. The antioxidant effect of SFN was also shown by Tarozzi et al. in SH-SY5Y neuroblastoma cells induced by hydrogen peroxide (H_2_O_2_) (300 μmol/L) or 6-OHDA (100 μmol/L) for 3 h. SFN (0.63–5 μmol/L) pretreatment increased the levels of GSH, GSH transferase, and NQO-1. Likewise, to evaluate the cytoprotective role of SFN, it was shown that treatment with buthionine sulfoximine inhibited the activity of GSH and also, consequently, the cytoprotective effects of SFN. Moreover, SFN treatment prevented necrosis and apoptosis by avoiding the activation of caspases 9 and 3, DNA fragmentation, and mitochondrial damage. Therefore, taken together, these results show that SFN could be useful to prevent the PD onset [90]. In order to reproduce specific characteristics of dopaminergic neurons and also in other cell lines, such as urothelial, human embryonic kidney cells, and human embryonic kidney that overexpress dopamine transporters as nondopaminergic cells, Shavali et al. investigated the antioxidant effects of SFN and N-acetylcysteine in SH-SY5Y neuroblastoma cells. To induce the experimental model, the authors used a mixture of arsenite (10 μM) and dopamine (100 μM) to induce the neurodegeneration of dopaminergic cells. It was shown that the pretreatment with SFN in the range (0.1–5 μM) for 24 h has significantly inhibited cell death induced by arsenite mixture and dopamine. In the same way, the incubation of N-acetylcysteine (100 μM, 1 mM, and 10 mM) with arsenite mixture and dopamine led to a reduction of cell death, according to a dose-dependent mechanism. Therefore, the antioxidant effect of SFN and N-acetylcysteine was mediated in part by intracellular events; in fact, SFN activating the dopamine-quinone reductase enzyme, protected SH-SY5Y cells by the viability loss. Similarly, N-acetylcysteine acting as a free radical scavenger through its thiol group improved GSH synthesis, reducing the oxidative stress, thus preserved from subsequent neuronal damage [91]. Therefore, the increase of GSH in dopaminergic neurons, mediated by Nrf2, may be a promising strategy for counteract the PD. In the case of the latter, Nrf2 activators like SFN and its metabolites such as erucin were investigated by researchers for their beneficial effects. Morroni et al. compared the effects of SFN and erucin both in vitro and in vivo. The SFN (5 μM) pretreatment in SH-SY5Y induced with 6-OHDA (100 μM), showed more increase in the active nuclear Nrf2 protein and also a more reduction of apoptosis compared to erucin (5 μM) treatment. These effects were also likely due to more ability of SFN to reduce DNA fragmentation in oligosomes. Instead, the simultaneous treatment of SFN and erucin at the same 5 μM dose, demonstrated similar protective effects, probably in the latter, SFN and erucin significantly enhanced the increase of nuclear protein Nrf2 and GSH levels. In order to confirm the results obtained in vitro, the authors performed the study in male C57Bl/6 mice administered unilaterally with a stereotaxic intrastriatal injection of 6-OHDA (2 μL). The study results showed that intraperitoneal treatment with SFN (30 μmol/kg) or erucin in the same dose, four weeks after the injury, improved the response in behavioral tests. In addition, the subsequent immunohistochemical analysis demonstrated that the treatment with both isothiocyanates reduced the loss of dopaminergic neurons and apoptosis [92] (see Table 3).

### 4.2. Effects of Sulforaphane in In Vivo PD Models

Morroni et al. also demonstrated the effects of SFN in male C57Bl/6 mice administered with an intrastriatal injection of 6-OHDA (2 μL). After four weeks of the model induction, the animals were treated with SFN (5 mg/kg) via intraperitoneal. SFN treatment improved the motor deficits 6-OHDA-induced, as well as protect the neurons in sustantia nigra from degeneration, as demonstrated by subsequent immunohistochemical analysis. In the same way, SFN by blocking DNA fragmentation and caspase-3 activation preserved neuronal cells from apoptosis. In addition, the neuroprotective role of SFN was evaluated through colorimetric analysis by the increase of GSH levels and its enzymes, and also through Nrf2 up-regulation and modulation of ERK 1/2 pathway. Therefore, the study showed that SFN, through modulation of oxidative stress and apoptosis, can be useful to counteract the progression of the disease [93]. Zhou et al. investigated the effects of SFN (50 mg/kg) administered via intraperitoneal in male C57BL/6 mice, induced orally with rotenone (30 mg/kg) administered for 60 consecutive days. The treatment with SFN prevented the motor deficits and dopaminergic neuronal loss induced by rotenone. Moreover, SFN through Nrf2 has been shown to reduce oxidative stress, evaluated through MDA and GSH levels. Furthermore, the Western Blot analysis demonstrated an increase both in the Nrf2 expression and antioxidant enzymes NQO-1 and HO-1. In addition, the treatment with rotenone induced cell death and dysregulated autophagy, by negatively regulating the mTor signaling pathway involved in cell metabolism, growth, proliferation, and survival. Therefore, SFN can exert its effects, activating the mTor signaling pathway, through its components, phosphorylation of p70 S6 kinase, and eukaryotic initiation factor 4E located downstream of it. Moreover, it was also shown that SFN can counteracting apoptosis by blocking the activity of caspase 3, which was increased after induction with rotenone. Besides, SFN can also modulate autophagy by blocking the reduction in the levels of LC3-II, reduced following treatment with rotenone [94]. Although the mechanisms underlying PD pathogenesis are not fully understood, environmental factors such as diet are also helpful to reduce the risk of the disease. Pu et al. investigated whether glucoraphanin, the precursor of SFN, administered as a food pellet can influence the loss of dopaminergic neurons in the corpus striatum. Male C57BL/6 mice were fed with glucoraphanin-enriched pellet or normal pellet for 28 days. In order to induce the experimental model, the animals were infused via intraperitoneal with MPTP (10 mg/kg) and compared to the control animals. The study results demonstrated that the treatment with 0.1% glucoraphanin pellet preserved the dopaminergic neurons from neurodegeneration, evaluated through the immunoreactivity for tyrosine hydroxylase [95]. In addition, Jazwa et al., in the MPTP mouse model induced via intraperitoneal with MPTP (30 mg/kg) for five consecutive days, showed the neuroprotective effects of SFN (50 mg/kg) administered via intraperitoneal. The study demonstrated that SFN, by acting through Nrf2, attenuated both the nigrostriatal neurodegeneration and neuroinflammation induced by MPTP. Indeed, SFN led to an increase in Nrf2 levels in the basal ganglia and the consequent upregulation of NQO-1 and HO-1, thus reducing the oxidative stress. The mechanism of action by which SFN exerts its effects was explained using Nrf2-KO mice. Indeed, in Nrf2-KO mice compared to wild-type mice, it was demonstrated that the treatment with SFN did not preserve the animals from the neurodegeneration of dopaminergic neurons and neither from the neuroinflammation, evaluated through the reduction of astrogliosis, microgliosis, and by release of proinflammatory cytokines. In addition, it was also observed that in the absence of activators, Nrf2 has a short half-life; in fact at the cytoplasmic level Nrf2, it forms a complex with the Keap1 protein that determines its consequent degradation through UPS. Instead, Nrf2 activators such as SFN induce its translocation into the nucleus and the subsequent transcription of phase II genes. Therefore, the effects of SFN as a potential neuroprotective inducer of enzymes and antioxidant molecules of phase II, could be useful for counteracting the disease [96] (see Table 4).

## 5. Effects of Sulforaphane in Experimental Studies of Multiple Sclerosis

MS is an autoimmune and demyelinating neurodegenerative disease that occurs mainly in females. The disease is characterized by several symptoms such as motor impairment, fatigue, pain, depression, visual problems, and bladder, intestinal, and sexual dysfunction [97]. MS shows a neuronal loss and subsequent neurological damage associated with infiltration of immune system cells, activation of microglia and progressive demyelination [98]. The demyelination allows cells of the immune system such as self-reactive T helper 1 and T helper 17 lymphocytes, and other immune cells, following the breakdown of the blood–brain barrier (BBB) to infiltrate. Indeed, the lymphocytes recognize the myelin antigen, cross the BBB, thus triggering a cascade of inflammatory events that lead to the lesion of the myelin sheath [99]. The myelin sheath involved in the transmission of electrical signals provides electrical isolation of the axons. Moreover, its production is related to oligodendrocytes, neuroglia cells located in the CNS. The oligodendrocytes, prior to producing myelin, undergo different stages of differentiation, starting from oligodendrocyte progenitor cells, which are generated during embryonic development in areas such as the subventricular zone, to migrate to the brain and spinal cord [100]. During maturation, the oligodendrocyte progenitor cells lose their proliferative and migratory capacity to assume morphological characteristics of mature oligodendrocytes [101]. The inflammatory state and the consequent loss of myelin determine the recruitment of precursors of oligodendrocytes, to differentiate into mature oligodendrocytes, and thus produce new myelin [102]. Although, demyelination may be followed by the myelin repair in which oligodendrocytes remyelinate axons to re-establish nerve impulses in the CNS [103]. It was also demonstrated that remyelination can occur an early phase of the MS [104]. Moreover, it was also shown that myelin repair is mediated by inflammation; in fact, the lesion allows the stimulation and recruitment of oligodendrocyte progenitor cells, capable of differentiating into mature oligodendrocytes to form the myelin sheath in axons [105,106]. Although several factors contribute to the etiopathogenesis of disease, both neuroinflammation and oxidative stress appear to be directly involved in the damaging events that occur to it. Indeed, it was shown that the pathogenesis of the disease can involve the oxidative stress through the uncontrolled production of ROS and RNS, which in turn also favor the progression of inflammation [107,108,109]. In addition, the oxidative stress can lead to demyelination and neurodegeneration directly through lipid, protein, and DNA oxidation, but also indirectly, inducing a proinflammatory response favored by immunity dysregulated [110,111]. Therefore, it was shown that the Nrf2/ARE signaling pathway, whose role as a modulator of antioxidant and phase II detoxification genes, may be a useful therapeutic target for MS [112]. Indeed, the activation of the Nrf2/ARE pathway can protect against tissue damage mediated by the activation of proinflammatory mediators. In particular, it was shown that Nrf2 KO in experimental autoimmune encephalomyelitis (EAE) mice promotes the neuroinflammation evaluated by the increase of interferon-γ (IFN-γ) and IL-17 and also gets worse the disease [113]. Likewise, Nrf2 activators such as SFN can modulated positively the Nrf2/ARE pathway, thus preserving the neuroinflammation [114]. Therefore, SFN, by modulating the antioxidant response can be useful for counteracting the harmful events such as oxidative stress and neuroinflammation that characterize the MS [115].

### 5.1. Effects of Sulforaphane in In Vivo MS Models

Several experimental studies demonstrated the effects di SFN in MS. Li et al. showed, in female EAE C57Bl/6 mice induced subcutaneously with myelin oligodendrocyte glycoprotein peptide 35–55 (250 μg), the anti-inflammatory effects of SFN, as well as the mechanisms underlying SFN action in the development and progression of the disease. The treatment intraperitoneal with SFN (50 mg/kg) after 48 h by model induction, inhibited the development and progression of the MS. Indeed, SFN upregulated the Nrf2/ARE pathway, increased the expression levels of HO-1 and NQO-1, leading to a reduction of oxidative stress. In addition, SFN inhibited the inflammation induced by T cells, also increasing the levels of anti-inflammatory cytokine IL-10. Moreover, SFN protected the integrity of BBB, as shown by tight junction proteins occludin and claudin-5 levels, as well as by the reduction in the expression levels of matrix metallopeptidase 9, involved in the degradation of the extracellular matrix [116]. In addition, Yoo et al. investigated the anti-inflammatory effects of SFN in EAE C57Bl/6 mice induced with myelin oligodendrocyte glycoprotein peptide 35–55. The pre-treatment for 14 days with SFN (50 mg/kg/day), via intraperitoneal before the induction of the model, led to an improvement in behavioral deficits compared to the control mice. Moreover, it was shown that SFN reduced both the infiltration of inflammatory cells and demyelination mediated by T cells in the spinal cord. In addition, SFN treatment reduced the oxidative stress, as evidenced by the reduction of NO levels. Therefore, SFN can be useful in the prevention and progression of neuroinflammatory diseases such as MS [117] (see Table 5).

### 5.2. Effects of Sulforaphane in In Vitro MS Models

Since demyelination determines the activation of inflammatory cells, such as macrophages and microglia, that release cytotoxic mediators like ROS and TNF-α, with a consequent loss of oligodendrocytes and axonal damage, strategies useful to counteracting the neuroinflammation and neurodegeneration are necessary [118,119]. Lim et al. demonstrated the effects of SFN (5 µM), monomethyl fumarate (90 µM), and protandim (30 µg/mL), an herbal compound, in OLN-93 cells, a permanent oligodendrocyte cell line derived from primary glial cultures of rat. The authors showed that SFN, monomethyl fumarate, and protandim led to a reduction of oxidative stress induced by hydrogen peroxide and evaluated by the increase of HO-1, NQO-1, and p62 levels. Moreover, the pre-treatment with SFN and protandim prevented cell death of oligodendrocyte, induced by hydrogen peroxide. In addition, protandim through the reduction of ROS levels demonstrated a more effective in the differentiation of progenitor cells of oligodendrocytes, thus confirming its neuroprotective effects [120]. In order to investigate the potential anti-inflammatory and detoxifying properties of Nrf2 activators, Wierinckx et al. demonstrated the effects of SFN (1, 5, or 15 μM) and dimethyl fumarate (5, 15, or 30 μM) in primary co-cultures of astroglial and microglial cells of rats induced with lipopolysaccharide (100 ng/mL). The treatment with SFN or dimethyl fumarate reduced the inflammation evaluated by the production and release of TNFα, IL-1β, and IL-6. In addition, SFN and dimethyl fumarate have also shown to increase the NQO-1 activity and GSH levels. In conclusion, both SFN and dimethyl fumarate reduced the neuroinflammation and also enhanced the detoxifying activity of astroglial and microglial cells induced by lipopolysaccharide [121] (see Table 6).

## 6. Conclusions

The aim of this review is to provide an overview of experimental studies that described the efficacy of SFN in the main neurodegenerative diseases including AD, PD, and MS. The results of the studies suggested that SFN through its molecular target Nrf2 can exert a beneficial role by activating genes and molecules with antioxidant, anti-inflammatory, and anti-apoptotic properties. Therefore, based on its effects, as well as a low neurotoxicity, SFN could be useful as a support to current therapies for the management of neurodegenerative diseases. Moreover, although some mechanisms are still not fully known, Nrf2 can be considered a useful therapeutic target for developing new strategies capable of counteracting the progression of neurodegenerative diseases such as AD, PD, and MS. However, further studies are needed to elucidate the molecular mechanisms underlying the properties of SFN and also to identify new molecular therapeutic targets.

## Figures and Tables

**Table 1:** Synthesis of the in vivo studies that evaluate the effects of sulforaphane (SFN) in the treatment of AD. Specifically, the table describes the models used, the type of treatment, the dosage, and the results obtained.

Experimental Models	Dose/Concentration Range	Route of Administration	Results	References
PS1V97L transgenic mice and primary cortical cells of rats	SFN (5 mg/kg) SFN (0.1 μM)	Intraperitoneal	In vivo, SFN improved cognitive deficits, inhibited Aβ aggregation and tau hyperphosphorylation, as well as reduced the oxidative stress and neuroinflammation. Instead, in vitro SFN improved cell viability and preserved dendritic length	[39]
Sprague-Dawley male rats	SFN (5 mg/kg)	Intraperitoneal	SFN improved depressive behaviors, reduced oxidative stress and neuroinflammation	[40]
C57BL/6 mice	SFN (25 mg/kg)	Oral	SFN treatment improved cognitive and motor deficits, reduced oxidative stress and formation of Aβ plaques both in the cortex and hippocampus	[41]
Kunming mice	SFN (25 mg/kg)	Gavage	SFN improved cognitive deficits as well as attenuated the loss of cholinergic neurons in the hippocampus and medial septum	[42]
ICR mice	SFN (30 mg/kg)	Intraperitoneal	SFN improved cognitive and memory deficits, as well as reduced the oxidative stress and prevented Aβ aggregation	[43]
3 × Tg-AD mice	SFN (10 or 50 mg/kg)	Gavage	SFN improved memory and learning deficits. Moreover, SFN reduced Aβ levels in the cortex, as well as Aβ and tau levels in the hippocampus	[50]
type 2 diabetes mellitus transgenic mice	SFN (1 mg/kg)	Intraperitoneal	SFN improved the cognitive deficits, it also reduced the oxidative stress and Aβ aggregation as well as phosphorylated tau levels in the hippocampus	[54]

SFN: sulforaphane; Aβ: beta-amyloid; AD: Alzheimer’s disease.

**Table 2:** This table shows the in vitro studies that reported the effects of SFN in the treatment of AD. Specifically, the table describes the models used in the experimental studies, the type of treatment, the dosage, and the results obtained.

Experimental Models	Dose/Concentration Range	Route of Administration	Results	References
SH-SY5Y cells and 5xFAD and 3 × Tg-AD mice	SFN (1 μM) SFN (5 or 10 mg/kg)	. Intraperitoneal	In vitro, SFN treatment led to a reduction in the amyloidogenesis and oxidative stress. While, in vivo SFN administration, improved cognitive deficits and reduced Aβ aggregation	[48]
SH-SY5Y cells	Crude juices of broccoli sprouts (10 μM)	.	The treatment with broccoli juices reduced cell death and oxidative stress Aβ-induced	[55]
SH-SY5Y cells	SFN (1 μM, 2 μM and 5 μM)	.	SFN protected cells from cytotoxicity and apoptosis induced by Aβ_25__–_35__. Moreover, the SFN treatment reduced oxidative stress	[56]
Microglial cells	SFN (5 µM)	.	SFN treatment improved microglia phagocytosis, reduced due to Aβ aggregates	[58]
Murine microglia cell BV2 and neuroblastoma cell N2a and male ICR mice	SFN-enriched broccoli sprouts 10 Μl Broccoli sprout (200 mg/kg)	. Oral	In vitro, SFN treatment reduced the inflammation, oxidative stress and apoptosis. While in vivo, the administration of SFN improved the memory deficits	[60]
Neuroblastoma N2a cells	SFN (1.25 and 2.5 μM)	.	SFN treatment decreased Aβ_1__–_40__ and Aβ_1__–_42__ levels in a dose-dependent manner. Moreover, SFN reduced the oxidative stress and the neuroinflammation	[61]
Human THP-1 macrophages	SFN (5 μM)	.	SFN treatment reduced neuroinflammation in Aβ_1__–_42__ induced macrophages	[62]
Human THP-1 macrophages	SFN (5 μM)	.	SFN through Mer tyrosine kinase could exert an anti-inflammatory effect induced by Aβ_1__–_42__	[64]
Cortical neurons of ICR mice and 3 × Tg-AD mice	SFN (10 or 20 Μm) SFN (10 or 50 mg/kg)	. Gavage	Both in vitro and in vivo, SFN treatment led to a reduction of neurodegeneration in the cortex and hippocampus	[65]
APP/presenilin1 double transgenic mouse and SH-SY5Y cells	SFN (25 mg/kg) SFN (2 μM)	Gavage .	In vivo SFN improved cognitive deficits and preserved the cortex from the increase of Aβ aggregates. While, in vitro, SFN prevented the reduction in cell viability	[71]

SFN: sulforaphane; Aβ: beta-amyloid; AD: Alzheimer’s disease; APP: amyloid precursor protein.

**Table 3:** We summarized in vitro studies that assess the effects of SFN in the treatment of PD. Specifically, we described the models utilized, the dosage, the type of administration, and the outcomes.

Experimental Models	Dose/Concentration Range	Route of Administration	Results	References
SH-SY5Y cells and Mouse embryonic fibroblasts	SFN (5 µM)	.	SFN reduced the oxidative stress and cell death	[79]
PC12 cells	SFN (0.5, 1.0, 2.5, 5.0 and 10 µmol/L)	.	SFN pre-treatment reduced cell damage induced by oxidative stress	[80]
PC12 cells	SFN (0.1, 1 and 5 µM)	.	SFN led to an increase in cell viability and inhibited cell death. In addition, SFN also reduced stress in the endoplasmic reticulum	[81]
PC12 cells	SFN (1 and 5 µM)	.	The pre-treatment with SFN prevented cell damage induced by 6-OHDA	[82]
dopaminergic neurons of organotypic rat nigrostriatal cultures	SFN (5 μM)	.	SFN and tert-butylhydroquinone reduced nigrostriatal neurodegeneration	[84]
PC12 cells and primary mesencephalic cultures	TPNA10168 (0.1–30 µM) and SFN (0.1–10 µM)	.	Both TPNA10168 and SFN treatment, protected dopaminergic neurons from neurodegeneration	[85]
primary cortical neurons of mouse	SFN (0.01–1 μM)	.	SFN pre-treatment protected neurons both from cell death and oxidative stress	[86]
CATH.a and SK-N-BE (2) C and mesencephalic dopaminergic neurons	SFN (0.5, 1, 2.5 and 5 μM)	.	SFN preserved neurons from neurodegeneration, reducing oxidative stress and favouring the increase of NQO-1 activity	[89]
SH-SY5Y cells	SFN (0.63–5 μmol/L)	.	SFN pre-treatment reduced oxidative stress and prevented necrosis and apoptosis	[90]
SH-SY5Y cells and also in urothelial, human embryonic kidney cells	SFN (0.1–5 μM)	.	The treatment with SFN and N-acetylcysteine reduced oxidative stress and consequent neuronal damage induced by the mixture of arsenite and dopamine	[91]
SH-SY5Y cells and C57Bl/6 mice	SFN (5 μM) SFN (30 μmol/kg)	. Intraperitoneal	In vitro, SFN pre-treatment increased the cell survival. While, in vivo, SFN improved behavioral deficits, reduced the loss of dopaminergic neuron and apoptosis	[92]

SFN: sulforaphane; 6-OHDA: 6-hydroxydopamine; NQO-1: nicotinamide adenine dinucleotide phosphate quinone oxidoreductase-1.

**Table 4:** The table summarizes in vivo studies that evaluate the effects of SFN in PD. In particular, we described the models utilized, the dosage, the type of administration, and the results.

Experimental Models	Dose/Concentration Range	Route of Administration	Results	References
C57Bl/6 mice	SFN (5 mg/kg)	Intraperitoneal	SFN administration improved motor deficits and protected the neurons from neurodegeneration and apoptosis	[93]
C57Bl/6 mice	SFN (50 mg/kg)	Intraperitoneal	SFN treatment prevented the motor deficits and loss of dopaminergic neurons. Moreover, SFN reduced oxidative stress	[94]
C57Bl/6 mice	0.1% glucoraphanin pellet	Oral	The treatment with 0.1% glucoraphanin pellet preserved the dopaminergic neurons from the neurodegeneration	[95]
Wild-type mice and Nrf2-KO mice	SFN (50 mg/kg)	Intraperitoneal	In wild-type mice, SFN acting through Nrf2 attenuated both nigrostriatal neurodegeneration and neuroinflammation	[96]

SFN: sulforaphane; Nrf2: nuclear factor erythroid 2 related factor 2; KO: knockout.

**Table 5:** This table shows the effects of SFN in vivo models of MS. Herein, were reported the models used, dosages, the type of administration, and results.

Experimental Models	Dose/Concentration Range	Route of Administration	Results	References
EAE C57Bl/6 mice	SFN (50 mg/kg)	Intraperitoneal	SFN through its antioxidant action reduced oxidative stress and inhibited inflammation	[116]
EAE C57Bl/6 mice	SFN (50 mg/kg/day)	Intraperitoneal	SFN improved behavioral deficits, also favoring the reduction of oxidative stress and neuroinflammation	[117]

EAE: experimental autoimmune encephalomyelitis; SFN: sulforaphane; MS: multiple sclerosis.

**Table 6:** We reported the effects of SFN in vitro studies of MS. Moreover, were shown the models used, dosages, and outcomes.

Experimental Models	Dose/Concentration Range	Results	References
OLN-93 cells	SFN (5 µM)	The treatment with SFN, monomethyl fumarate and Protandim prevented oxidative stress induced by hydrogen peroxide	[120]
primary co-cultures of astroglial and microglial cells of rats	SFN (1, 5, or 15 μM)	SFN and dimethyl fumarate alone or in combination reduced inflammation and enhancing the detoxifying action	[121]

SFN: sulforaphane; MS: multiple sclerosis.

## Abbreviations

SFN	Sulforaphane
AD	Alzheimer’s disease
PD	Parkinson’s disease
MS	multiple sclerosis
ESP	epithiospecifier
GSH	glutathione
Nrf2	nuclear factor erythroid 2 related factor 2
NQO-1	nicotinamide adenine dinucleotide phosphate quinone oxidoreductase 1
HO-1	heme oxygenase 1
ARE	antioxidant response elements
Keap1	Kelch-like ECH associated protein 1
UPS	ubiquitin-proteasome system
Maf	musculoaponeurotic fibrosarcoma oncogene homologue
ROS	reactive oxygen species reactive
RNS	nitrogen species
KO	knockout
TNF-α	tumor necrosis factor-α
IL	interleukin
iNOS	inducible nitric oxide synthetase
COX-2	cyclooxygenase-2
MAPK	mitogen-activated protein kinase
NF-κB	nuclear factor kappa-B
ERK	extracellular signal-regulated kinase
JNK	c-Jun N-terminal kinase
LC3-II	light chain 3-II
ATP	adenosine triphosphate
BDNF	brain-derived neurotrophic factor
WNT	Wingless type
Aβ	beta-amyloid
APP	amyloid precursor protein
β-secretase1	BACE-1
MDA	malondialdehyde
HSP	heat shock protein
CHIP	C-terminus of HSP70-interacting-protein
BCL-2	B cell lymphoma-2
BAX	BCL2-Associated X
CNS	central nervous system
SOD	superoxide dismutase
p75^NTR^	p75 neurotrophin receptor
MPP^+^	1-methyl-4-phenyl pyridine
MPTP	1-methyl-4-phenyl-1,2,3,6-tetrahydropyridine
6-OHDA	6-hydroxydopamine
H_2_O_2_	hydrogen peroxide
PI3K/AKT	phosphatidylinositol-3-kinase
ASK1/MAP3K5	apoptosis signal-regulating kinase 1
BBB	blood-brain barrier
EAE	experimental autoimmune encephalomyelitis
IFN-γ	interferon-γ
